# Transcriptome-based maturation assessment revealed pro-maturation transcription factors of cardiomyocytes 

**DOI:** 10.3389/fbioe.2026.1717996

**Published:** 2026-02-18

**Authors:** Nawin Chanthra, Sean Murphy, Matthew Miyamoto, Nanami Masuyama, Nozomu Yachie, Chulan Kwon, Hideki Uosaki

**Affiliations:** 1 Division of Regenerative Medicine, Center for Molecular Medicine, Jichi Medical University, Shimotsuke, Japan; 2 Department of Biochemistry and Genetic Engineering, Faculty of Medicine, Kasetsart University, Bangkok, Thailand; 3 Division of Cardiology, School of Medicine, Johns Hopkins University, Baltimore, MD, United States; 4 School of Biomedical Engineering, Faculty of Applied Science and Faculty of Medicine, The University of British Columbia, Vancouver, Vancouver, BC, Canada; 5 WPI Premium Research Institute for Human Metaverse Medicine, Osaka University, Osaka, Japan; 6 Division of Functional Biochemistry, Department of Biochemistry, School of Medicine, Jichi Medical University, Shimotsuke, Japan

**Keywords:** cardiomyocytes, ERRα, maturation, PGC1, pluripotent stem cells, transcriptional factors

## Abstract

Induced pluripotent stem cell-derived cardiomyocytes have shown promise to be an essential tool for studying genetic cardiac diseases. However, their limited maturity remains a barrier to reaching their full potential. Many have challenged this problem; however, it is difficult to compare the results because the parameters for cardiomyocyte maturation are diverse, mostly relying on physiological experiments that display significant lab-to-lab variations and are labor-intensive, and are not comparable to maturing cardiomyocytes *in vivo*. Here, we propose a transcriptome-based scoring method for cardiomyocyte maturation. We first established the maturation score based on transcriptome of mouse ventricles from embryonic (day 11) to adult (10-month-old) ventricles. We then demonstrated that known maturation conditions increased the maturation scores of mouse embryonic stem cell-derived cardiomyocytes. We finally performed expression screening of 92 candidate transcriptional factors (TFs) and identified pro-maturation TFs, including peroxisome proliferator-activated receptor gamma coactivator 1-alpha (PGC1α), PGC1β, and estrogen-related receptor alpha (ERRα). These results support that the transcriptome-based maturation score is a quantitative and reliable approach for identifying pro-maturation factors for cardiomyocytes.

## 1 Introduction

Cardiomyocyte maturation progresses through fetal to adolescent stages (Yang et al., 2014a; Ahmed et al., 2022), and cardiomyocytes acquire key features such as developed sarcomeres, T-tubules, aligned mitochondria producing ATP through fatty acid oxidation. Understanding the molecular mechanism behind the cardiomyocyte maturation will lead better disease models with pluripotent stem cell-derived cardiomyocytes (PSC-CMs) that are considerably immature *in vitro*. While recent progress revealed several key molecules of cardiomyocyte maturation *in vivo* (Guo et al., 2018; Guo and Pu, 2020; Sakamoto et al., 2020; Murphy et al., 2021), the entire molecular mechanism has not been fully understood, including transcriptional regulation. We recently reported transcriptional factors (TFs) activated during the maturation process and aberrantly inactive TFs in mouse embryonic stem cell-derived cardiomyocytes (ESC-CMs) compared to mouse hearts (Uosaki et al., 2015). Here, we hypothesized that upregulation of one or a few of those TFs enhances cardiomyocyte maturation *in vitro*.

Although PSC-CMs form an essential platform for studying the maturation process of cardiomyocytes in more controlled and defined conditions (Nakano et al., 2017), analytical approaches of cardiomyocyte maturation remain to be refined and developed. Morphological, structural, or functional analyses are used to determine the maturity of PSC-CMs; however, these methods only determine relative maturity, not definitive maturity comparable to that of cardiomyocytes *in vivo*. The mentioned methods are also time-consuming and present lab-to-lab variations, which makes it more difficult to compare findings. Fluorescent reporters are an option, but are unable to capture the entire maturation process (Shinnawi et al., 2015; Chanthra et al., 2020; Chirikian et al., 2021; Miki et al., 2021; Maria Cherian et al., 2023). We recently demonstrated that a microarray-based transcriptome analysis can assess how mature mouse ESC-CMs are compared to mouse hearts, namely, MatStat^CM^ (Uosaki et al., 2015). MatStat^CM^ precisely determined the maturation stage of mouse ESC-CMs; however, it has limitations, *e.g.*, a limited time-resolution after birth, a time- and cost-consuming microarray-based method, and no human translational ability. In this study, we took advantage of the scalability of RNA-sequencing to overcome these shortcomings.

## 2 Methods

### 2.1 Mouse embryonic stem cell differentiation toward cardiomyocytes

In this study, a mouse embryonic stem cell (ESC) line, SMMB2, in which red fluorescent protein (RFP) was inserted in the endogenous *Myom2* locus and harboring a cardiac-specific puromycin-resistant gene, was used (Chanthra et al., 2020; Yamanaka et al., 2008). ESCs were differentiated toward cardiomyocytes (ESC-CMs) following the previously reported method (Uosaki et al., 2015; Chanthra et al., 2020; Ahmed et al., 2021). Briefly, the cells were cultured in suspension for the first 4 days of differentiation in Serum-free differentiation (SFD) medium, consisting of Ham’s F12, Iscove’s Modified Dulbecco’s Medium, B27 supplement minus Vitamin A, N2 supplement, GlutaMax, bovine serum albumin, ascorbic acid, 1-Thioglycerol, penicillin, and streptomycin. From day 2, Activin A, vascular endothelial growth factor (VEGF), and bone morphogenetic protein four were added. At day 4, cells were dissociated and replated to 0.1% gelatin-coated dish with basic fibroblast growth factor (FGF), FGF10, and VEGF. From day 7, puromycin was added to purify cardiomyocytes. ESC-CMs were replated on a 0.1% gelatin-coated dish or an extracellular matrix (ECM)-coated dish at day 10. For a specific case, Tri-iodo-I-thyronine (T3, 0.1 µM) and/or hypoxia-inducing factor 1α (HIF1α) inhibitor, Chetomin (10 nM), were used to enhance cardiomyocyte maturation from day 14–28 (Yang et al., 2014b; Gentillon et al., 2019). Myom2-RFP positive and negative cells were sorted on SH800 (SONY) at days 17, 24, and 38, respectively. For lentivirus transduction, a lentivirus vector-containing supernatant was applied at day 11. Culture media were replaced with SFD with and without T3 at day 14. At day 18, flow cytometry was conducted to examine Myom2-RFP, a maturation reporter, and GFP for lentivirus transduction efficiency. Then, the cells were subsequently collected for RNA-sequencing at day 21.

### 2.2 Lentivirus production

In this study, 92 TFs were tested. Most of TFs were from ORFeome or Ultimate ORF, but some were obtained from Addgene and RIKEN BRC, or cloned from mouse heart cDNA (Supplementary Table S1). We cloned each TF into the pLenti-6 vector using the Gateway system (Thermo Fisher). HEK293T cells were cultured with 10% fetal bovine serum-supplemented Dulbecco’s Modified Eagle Medium. For small-scale lentivirus production, we plated HEK293T cells in a 24-well plate, then psPAX2, pMD2.G, and each transfer vector (1 TF/w) were transfected on the following day. Culture medium was refed with 500 µL of the HEK293T culture medium 1 day after the transfection, and virus-containing supernatants were collected 3 days after the transfection. The supernatants were then centrifuged at 2000 rpm for 5 min to remove debris and floating HEK293T cells. 200 μL of the virus-containing supernatant was added to the ESC-CM culture (total 700 µL). We simultaneously transduced the GFP-encoded lentivirus into HEK293T cells to calculate the physical titer. Based on the titer, the MOI was 15–60 IFU/cell to ESC-CMs, and 30%–70% of ESC-CMs were GFP-positive 7 days after transduction. While we prepared additional GFP-encoded lentiviruses in separate wells to test transduction variability in a single set, we observed acceptable variability (52.6% ± 4.1%).

### 2.3 Animals

Ventricles and atria were excised from C57BL6/J mice at embryonic day 11 (E11) to postnatal day 56 (P56), and at 30 weeks and 10 months of age. Mice were purchased from Japan SLC, Inc. The excised hearts were homogenized in TRIzol solution for RNA isolation. All animal works were performed in accordance with the guidelines of the Institutional Animal Care and Concern Committee at Jichi Medical University and were approved by the committee.

### 2.4 Human samples

Human heart RNA samples were purchased from BioChain (Male, 24 years old, Cat #R1234122-50, Lot #B604038), Takara (Pooled, three males, 30–39 years old, Cat #636532, Lot #1702013A), and Clontech (Pooled four male fetuses, 24–30 weeks, Cat #636583, Lot #1412043).

### 2.5 RNA sequencing and analysis

RNA was purified using Direct-zol 96 RNA isolation plates (Zymo Research). Then, 500 ng RNA was used for the input of QuantSeq 3′mRNA-seq Library prep kit (Lexogen). Each library concentration was measured with a Qubit 4 fluorometer (Thermo Fisher Scientific), and 48 to 96 libraries were pooled to sequence for 75 cycles (single-end) on NextSeq (Illumina). Prior to sequencing, the library size was measured with BioAnalyzer 2100(Agilent) or MultiNA (Shimadzu).

RNA-sequence data was processed as previously reported (Chanthra et al., 2020). Briefly, the obtained sequence reads were first trimmed with BBDuk, then mapped to the GRCm38 mouse genome using STAR aligner (Dobin et al., 2013). FeatureCounts in Subread package was used to count the mapped reads at the gene level (Liao et al., 2014). We eliminated the samples with fewer than one million reads. Further analysis was performed on R (version 4.5.0), and graphs and plots were visualized with ggplot2 (ver 3.5.2) or tidyplots packages (Engler, 2025). For principal component analysis (PCA) or maturation score calculation, we excluded low-expressing genes (average transcript per million is less than one among mouse heart samples).

### 2.6 Maturation score calculation

The maturation scores were calculated following the equation. ω_i_ is the weight of the *i*th gene for principal component (PC) one of ventricular RNA-seq data, while ε_i_ is the expression (transcript per million) of the *i*th gene of a given sample. The equation was empirically made to convert PC1 score values to approximately a 0–100 scale. In the equation, $\sum_{i=1}^{n}\left(\omega_{i}\times \log\left(\varepsilon_{i}+1\right)\right)$ is a PC1 value. Given that the PC1 values of ventricles range between −32 and +33.5, 32 was added to the PC1 value and then multiplied by 1.5.

$$Maturation\;Score=1.5\times \left(32+\sum_{i=1}^{n}\left(\omega_{i}\times \log\left(\varepsilon_{i}+1\right)\right)\right)$$

### 2.7 Humanized maturation score calculation

To harmonize the mouse and human transcriptomes, the dataset was reduced to 12,219 orthologs sharing gene symbols in mouse and human following the calculation of transcripts per million reads. Given that the PC1 values of mouse ventricles with the selected genes range between −26.3 and +26.9, 27 was added to the PC1 value and then multiplied by 1.8.

$$Humanized\;Maturation\;Score=1.8\times \left(27+\sum_{i=1}^{n}\left(\omega_{i}\times \log\left(\varepsilon_{i}+1\right)\right)\right)$$

### 2.8 Gene ontology analysis

Highly weighted genes to PC1 (weight more than mean ±2 x SD) were selected for gene ontology analysis. Gene list was submitted to PANTHER Overrepresentation Test (Released 20240807) at https://geneontology.org/ (Ashburner et al., 2000; Thomas et al., 2022; Aleksander et al., 2023).

### 2.9 Statistics

Statistics were performed with R. For multi-group comparison, Tukey honestly significant difference (HSD) or Wilcoxon test was used depending on the comparison, and P values less than 0.05 were considered significant. Both tests were called from rstatix (ver 0.7.2) *via* tidyplots (ver 0.2.2) on R. We did not perform any statistics on the sample numbers less than 3.

### 2.10 Data availability statement

The RNA-seq datasets for this study can be found in the DDBJ Sequence Read Archive (DRA) under the accession number PRJDB39923. Code to reproduce figures and graphs is attached as Supplementary Material.

## 3 Results

### 3.1 Chronological RNA-sequence of mouse hearts

**FIGURE 1:**
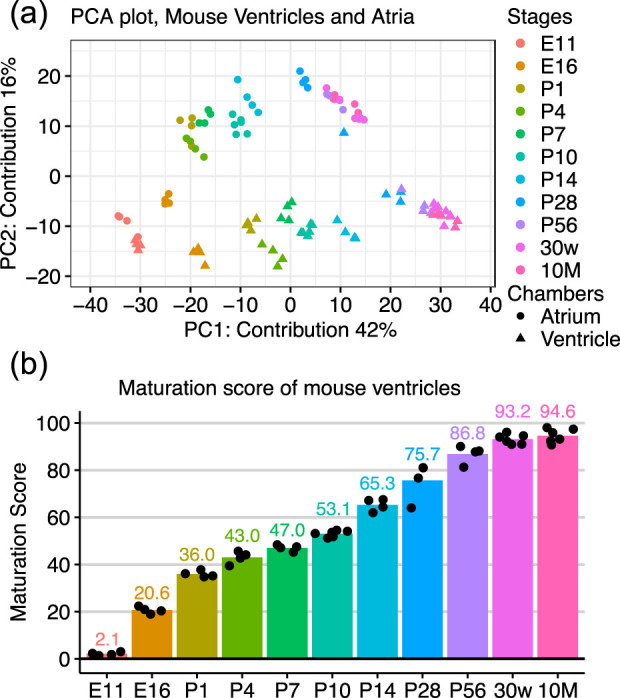
Maturation Scores of Mouse Hearts **(a)** Scatter plot of PCA of RNA-sequence data of mouse ventricles and atria obtained from E11 to 10-month-old mice. **(b)** PC1 values of ventricles were converted to a 0–100 scale, namely, Maturation Score. Four to six independent samples were analyzed.

We recently identified TFs that are aberrantly dysregulated in ESC-CM culture and/or activated during CM maturation in the mouse heart (Uosaki et al., 2015). Here, we hypothesized that overexpression of these inactive TFs enhances maturation in ESC-CMs. To test this idea, we needed a middle-to-high-throughput approach to determine ESC-CM maturity, as the candidates were 92 TFs. We previously reported that the transcriptome dataset can be used as a good measure for cardiomyocyte maturation (Uosaki et al., 2015), however, the MatStat^CM^ method relied on a microarray platform, which is not appropriate for our purpose. Moreover, it lacked time resolution between neonate and adult, a critical stage for cardiomyocyte maturation. Therefore, we first determined to develop an RNA-sequence-based method for cardiomyocyte maturation with higher time resolution. We selected QuantSeq 3′mRNA-seq as the library preparation method because it is cost-effective compared to total mRNA-sequencing. We performed a chronological RNA-sequence of mouse ventricles and atria using QuantSeq at relevant timepoints to generate a reference atlas. We found maturation-related changes on PC1 axis while PC2 axis segregated atria from ventricles (Figure 1a). This result was consistent with previous findings (Uosaki et al., 2015; Anzai et al., 2020). Because the reference atlas was generated from whole ventricles and atria, rather than isolated cardiomyocytes, we examined the genes contributing most strongly to PC1. The top weighted genes included *Ckmt2*, *Mb*, and *Xirp2*, all of which are well characterized for their roles in mitochondrial energy metabolism, cardiomyocyte structure, and sarcomere function. In contrast, *Tnni1*, known to switch to *Tnni3* in adult ventricles, led the negatively contributed genes. Gene ontology (GO) analysis was further performed on positively weighted genes to the PC1 axis (Supplementary Table S2). GO-cellular component (CC) revealed that mitochondria and sarcomere-associated genes were highly enriched. Furthermore, many of the enriched GO-molecular function (MF) and biological process (BP) terms for positively weighted genes to the PC1 axis were related to oxidative phosphorylation, including fatty acid metabolic process in GO-BP, suggesting that those genes were most likely related to cardiomyocyte function. In contrast, terms for negatively weighted genes to the PC1 axis were cell cycle regulators (GO-BP) localized in the chromosome (GO-CC), consistent with the fact that cardiomyocytes leave the cell cycle postnatally. Finally, we aimed to make the results easier to read. Thus, we empirically converted the PC1 axis to a 0–100 scale (Figure 1b), providing a linearly increasing score throughout the cardiomyocyte maturation process.

### 3.2 Maturation scores of ESC-CMs in various pro-maturation conditions

**FIGURE 2:**
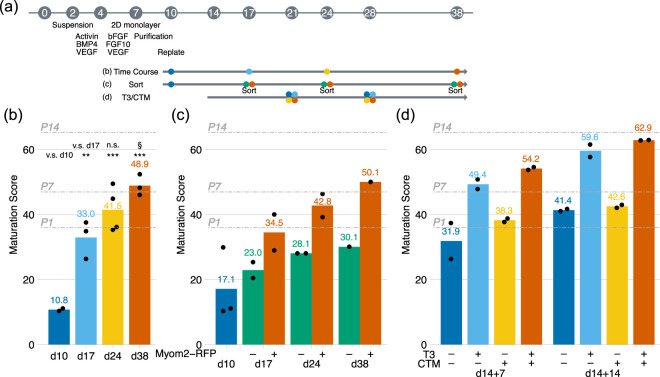
Maturation Scores of Mouse ESC-CMs **(a)** Schema of cardiac differentiation and maturation experiments. **(b)** Maturation scores of mouse ESC-CMs on 0.1% gelatin for the extended culture. Tukey HSD tests were used. *, §, p < 0.05; **, p < 0.01; ***, p < 0.001. N = 2 for day 10, while three to four biological replicates were used. **(c)** Maturation scores of Myom2-RFP positive or negative cells at days 17, 24, and 38, respectively. N = 3 for day 10, while two biological replicates were used except for day 38. **(d)** Maturation scores of mouse ESC-CMs with T3 and CTM treatments at days 21 and 28. The treatment was started on day 14. Two biological replicates were used. Maturation scores of P1, P7, and P14 ventricles are shown as gray dashed lines.

Next, we aimed to test whether the maturation score worked for mouse ESC-CMs. We tested three different conditions (Figure 2a)—extended culture period (Kamakura et al., 2013), Myom2-RFP sorted cells (Chanthra et al., 2020), and thyroid hormone (Yang et al., 2014b) combined with inhibition of hypoxia-inducing factor 1α (HIF1α) (Gentillon et al., 2019). The maturation scores of ESC-CMs were significantly increased during the extended culture periods from those of E11 and E16 ventricles to those of post-natal, P7 to P10, ventricles by day 38 (Figure 2b), suggesting a slower maturation process of ESC-CMs compared to that of the heart (28 days *in vitro* vs. 9–18 days *in vivo*). We also sorted Myom2-RFP positive and negative cells and found that the maturation scores of Myom2-RFP positive cells were consistently higher than negative cells (Figure 2c). These results were comparable to our previous report (Uosaki et al., 2015), supporting the feasibility of the method. Thyroid hormone is one of the most studied pro-maturation supplements (Yang et al., 2014b). Postnatal switch from hypoxia to normoxia induces cell cycle exit in cardiomyocytes and a metabolic switch from glycolysis to fatty acid oxidation (Nakada et al., 2017; Ahmed et al., 2020). HIF1α is a key molecule to sense hypoxic conditions. Moreover, our previous analysis identified that HIF1α was activated in ESC-CMs *in vitro* compared to *in vivo* hearts (Uosaki et al., 2015). Chetomin (CTM) is a specific inhibitor of HIF1α (Kung et al., 2004). Here, we tested the combination of thyroid hormone (T3) and CTM. As expected, T3-treatment increased the maturation scores at days 21 and 28. In contrast, CTM alone displayed very weak effects on the maturation (Figure 2d). To ask if transcriptome results were comparable with other maturation parameters, we evaluated the morphology of ESC-CMs (Supplementary Figure S1). ESC-CMs elongated during the first 7 days of observation (from day 14–21), which was consistent with the maturation score. With T3 treatment, ESC-CMs became thicker and developed more aligned sarcomeres, supporting the progression of maturation. We also examined the expressions of the previously reported maturation-related genes (Supplementary Figure S2) (Anzai et al., 2020). Upon T3 or T3+CTM treatments, *Tnni1* to *Tnni3* and *Myh7* to *Myh6* isoform switches were observed. Interestingly, *Myom2* was downregulated, which was consistent with Myom2-RFP results. Metabolic genes (*Cox7a1*, *Ckmt2*, and *Mb*) and calcium-handling genes (*Atp2a2*, *Pln*, and *Ryr2*) were upregulated more than two-fold, supporting that ESC-CMs with T3 or T3+CTM treatments were at more mature stages.

### 3.3 Transcriptional factor upregulation improved cardiomyocyte maturation

**FIGURE 3:**
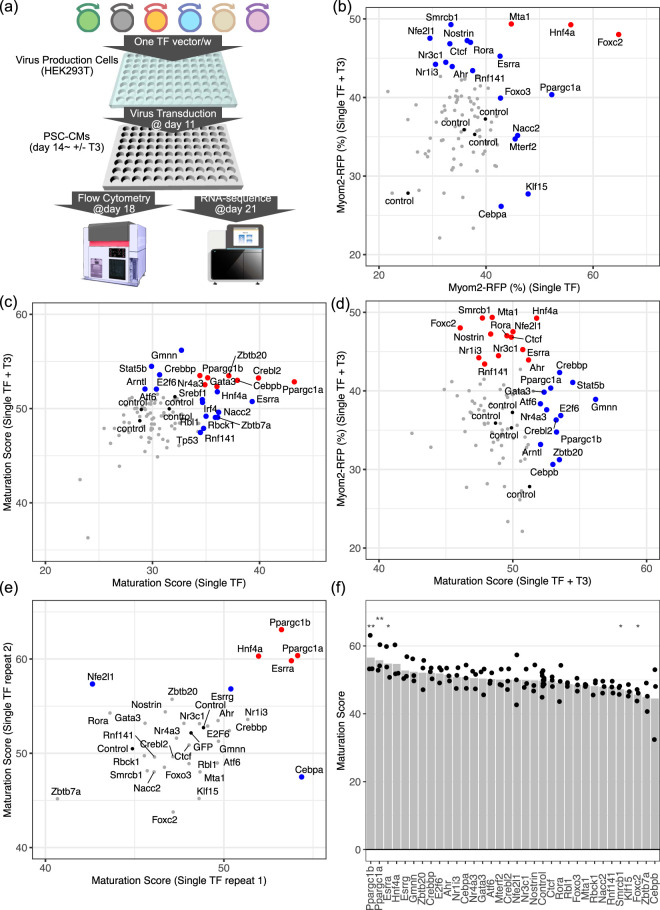
Pro-maturation Transcriptional Factors **(a)** Schema of lentivirus transduction and cardiac maturation experiments. **(b,c)** Evaluation of cardiomyocyte maturation after lentivirus transduction. **(b)** Myom2-RFP and **(c)** Maturation scores were examined with and without T3 at days 17 and 21, respectively. Red dots indicate Myom2-RFP (%) or the maturation scores were >mean + SD in both experiments with or without T3, while blue dots indicate >mean + SD in only one experiment. **(d)** Maturation scores and Myom2-RFP (%) with T3. Blue and red dots represent TFs selected by the maturation score and Myom2-RFP, respectively. **(e)** Scatter plot of the maturation scores of mouse ESC-CMs transduced with top TF candidates for cardiomyocyte maturation in two additional replicates. **(f)** Bar plot of the maturation scores of mouse ESC-CMs transduced with top TF candidates for cardiomyocyte maturation in two additional replicates and the initial screening. Control included no insert and GFP-coding vectors (Four controls in the screening; three controls in an additional replicate). Wilcoxon test was used. *, p < 0.05; **, p < 0.01 vs. control.

We tested whether overexpression of TFs promotes ESC-CM maturation. To this end, we selected 92 TFs from our previous study (Supplementary Table S1) (Uosaki et al., 2015). TFs were selected based on (1) their lower activity in ESC-CMs compared to neonate or adult hearts, (2) increased activity in adult hearts, or (3) core TFs in the transcriptome network of adult hearts. For the initial screening, we examined single TF transduction using lentivirus vectors, then examined Myom2-RFP and the maturation scores with and without T3 (Figures 3a–c). 19 TFs increased Myom2-RFP either with or without T3 (Figure 3b), while 23 TF increased the maturation scores (Figure 3c). Interestingly, only 5 TFs increased both Myom2-RFP and the maturation score, and most of them were discrete (Figure 3d).

Here, we selected 33 TF candidates from both analyses for further analysis. Since the maturation score accounts for both Myom2 and other maturation-related gene expressions, we repeated the maturation score analysis with the top TF candidates (Figures 3e,f). Repeated measurements identified peroxisome proliferator-activated receptor gamma coactivator 1-alpha (Ppargc1a, also known as PGC1α), Ppargc1b (PGC1β), estrogen-related receptor alpha (Esrra; ERRα), and hepatocyte nuclear factor 4 alpha (HNF4α) displaying higher maturation scores. Among them, PGC1α, PGC1β, and ERRα significantly increased the maturation scores to approximately 55 or more by day 21, corresponding to approximated P10 to P14 ventricles, while that of T3-treated control ESC-CMs was 50 (P7 to P10), when combining three independent experiments, including the initial screening (Figure 3f). Most importantly, the top 3 TFs, PGC1α, PGC1β, and ERRα, were known regulators of cardiomyocyte maturation (Sakamoto et al., 2020; Murphy et al., 2021; Miki et al., 2021), supporting the feasibility of the maturation score.

We further evaluated the transgene expressions and their downstream effects the transcriptome dataset. First, we examined whether transgenes were upregulated (Supplementary Figure S3). To this end, we selected 12 TFs showing higher maturation scores. We confirmed significant upregulation in *Ppargc1b*, *Esrra*, *Esrrg*, *Gmnn*, *Ahr*, and *Cebpa*. *Hnf4a* was also tended to be upregulated. In contrast, *Ppargc1a* was not upregulated. Although the detailed reason is still unclear, we considered that this was due to a mapping issue of the transgene to endogenous genes. Moreover, *Gmnn* was upregulated by TFs with higher maturation scores, such as PGC1α, PGC1β, ERRα, and HNF4α, whereas *Cebpa* was upregulated by HNF4α to a similar degree as CCAAT/enhancer-binding protein (Cebpa, C/EBPα) transgene. Next, we evaluated maturation-related genes (Supplementary Figure S4a-c). (Anzai et al., 2020). We considered that genes with P < 0.05 and more than two-fold changes were significant. Among maturation-related sarcomeric genes, *Myh7* was decreased by PGC1β, while *Tcap* was upregulated by ERRα (Supplementary Figure S4a). Moreover, several metabolic genes associated with cardiomyocyte maturation, including *Ckmt2*, *Cox7a1*, and *Fabp3*, were upregulated (Supplementary Figure S4b). In contrast, Maturation-related calcium-handling genes were relatively unchanged (Supplementary Figure S4c). To further investigate the global metabolic genes, we selected more than 200 genes listed in oxidative phosphorylation hallmark at the Molecular Signatures Database (MSigDB) and found that PGC1α and PGC1β significantly upregulated overall gene expressions related to oxidative phosphorylation (Supplementary Figure S5).

### 3.4 Translation to human models

**FIGURE 4:**
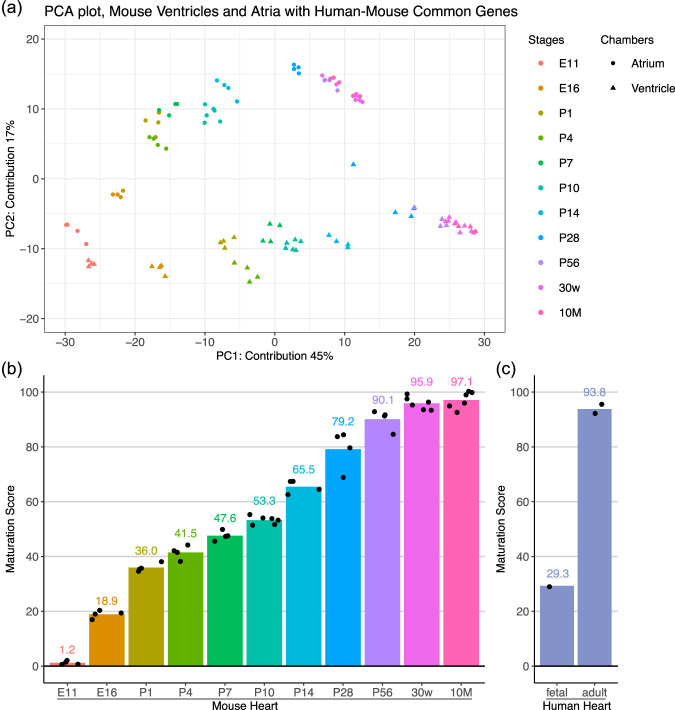
Translation to Human Models **(a)** Scatter plot of PCA of RNA-sequence data of mouse ventricles and atria obtained from E11 to 10-month-old mice. PCA was performed only with the common genes between mouse and human. **(b)** PC1 values of ventricles with the common genes between mouse and human were converted to a 0–100 scale, namely, Humanized Maturation Score. **(c)** Humanized maturation scores of fetal and adult hearts.

A comprehensive understanding of human cardiomyocyte maturation ideally require datasets spanning all developmental stages from fetus to neonate to adult hearts. However, such datasets are limited due to ethical and practical constraints (Cardoso-Moreir et al., 2019). Therefore, we aimed to ask if our method can be extended to human cardiomyocyte analysis. First, we selected mouse and human orthologs sharing the same gene symbols and performed principal component analysis (PCA) on mouse heart samples using these genes. The overall structure remained similar to the original PCA plot with the full gene set (Figures 1a, 4a). Then, we re-established “humanized” maturation scores from the PC1 axis with mouse and human common genes (Figure 4b). Finally, we examined human hearts at two different stages to ask whether the humanized maturation score can be used to evaluate human cardiomyocyte maturation (Figure 4c). The maturation score of pooled fetal hearts (24–30 weeks) was close to E16–P1 mouse ventricles, while those of adult hearts (24–39 years) were at P56–30w mouse ventricles. These results supported our idea of the humanized maturation score.

## 4 Discussion

### 4.1 Advantages of transcriptome-based method for determining cardiomyocyte maturation

In this study, we updated a transcriptome-based scoring method for cardiomyocyte maturation with RNA-sequence and demonstrated that the maturation score identified known pro-maturation TFs from 92 candidate TFs. While maturation reporters such as Myom2-RFP present a significant advantage with their simpler experimental flow, the maturation score demonstrated higher reproducibility and more accurate indication. Myom2-RFP positive cells displayed mature phenotypes and higher maturation scores, suggesting Myom2-RFP is a good indicator of cardiomyocyte maturation (Figure 2c) (Chanthra et al., 2020). However, T3 treatment did not increase Myom2-RFP positive fraction. Furthermore, PGC1α, PGC1β, and ERRα weakly increased Myom2-RFP. In contrast, the maturation scores were increased upon T3 treatments. Compared to the reliance on a single gene in the maturation reporters, the maturation score accounts for the entire transcriptome; therefore, it is more resilient to perturbations.

### 4.2 Potential translation to human cardiomyocyte maturation

In the present study, we demonstrate that a maturation score originally derived from mouse ventricles can be adapted for use with human heart samples. Our previous analysis indicated that mouse hearts at P0–3 correspond to human hearts at approximately 19 weeks of gestation, as inferred from a re-examination of the publicly available dataset (Anzai et al., 2020). Due to constraints in commercially available human cardiac RNA, we were limited to generating the humanized maturation scores from pooled fetal samples (26–30 weeks of gestation) and adult specimens (one individual aged 24 years and a composite pool ranging from 30 to 39 years). The resulting scores aligned with mouse embryonic (E16–P1) and young adult (P56–30w) ventricles for the human fetal and adult hearts, respectively, indicating that the humanized maturation score yields biologically plausible estimates.

It is well established that several key cardiac genes undergo isoform switching during the embryonic-to-adult transition, and that a subset of these switches proceeds in opposite directions in mouse versus human (Ahmed et al., 2022). We mapped orthologous transcripts by matching gene symbols shared between the mouse and human transcriptomes to establish cross-species correspondence in this study. Although incorporating isoform information could theoretically improve cross-species correspondence, this straightforward symbol-level mapping proved sufficient for the objectives of the present study.

### 4.3 Limitation and future directions

The top TFs identified in this study, including PGC1α, PGC1β, and ERRα, are known for their roles in cardiomyocyte maturation (Sakamoto et al., 2020; Murphy et al., 2021; Miki et al., 2021), which validates the maturation score as a method of determining the maturity of PSC-CMs. Since the roles of the top hits are known, we did not intensively validate further with cellular morphology, structure, and function, which should be noted as the limitation of this study (Ahmed et al., 2020). Moreover, we had an aim to combine these TFs to test if further maturation could be observed; however, we were unable to achieve high transduction, which precluded us from testing the combinatorial effects of the TFs.

During the postnatal maturation process, metabolism-related genes are highly upregulated, while cell cycle genes are downregulated in mice, humans, or opossums (Uosaki et al., 2015; Anzai et al., 2020; Uosaki and Taguchi, 2016; Nishiyama et al., 2022). Indeed, the top and bottom weighted genes that convert into the maturation score were related with these aspects more than other maturation-related changes (Supplementary Table S2). Generating a subset of maturation-related genes from a gene set and/or gene ontology for a specific cardiac function (e.g., sarcomere) will assist in overcoming the limitation and uncovering transcriptional regulations of the cardiomyocyte maturation process.

In this study, we focused on ventricular maturation as it is in the best interests of the field. On the other hand, atrial differentiation and cardiac organoid research have gained traction (Andersen et al., 2018; Moriwaki et al., 2023). On PCA plot (Figure 1a), atrial maturation started from a similar point with the embryonic ventricles, and both PC1 and PC2 values increased while maturing. Moreover, the PC1 values of adult atria were less than those of adult ventricles, suggesting the maturation score may underscore the maturation of atrial cardiomyocytes. However, we may be able to pinpoint the extent of maturation and atrial/ventricular lineage specification simultaneously using the entire PCA or transcriptome.

## Data Availability

The RNA-seq datasets for this study can be found in the DDBJ Sequence Read Archive (DRA) under the accession number PRJDB39923. Code to reproduce figures and graphs is attached as Supplementary Material.

## References

[B1] Ahmed R. E. Anzai T. Chanthra N. Uosaki H. (2020). A brief review of current maturation methods for human induced pluripotent stem cells-derived cardiomyocytes. Front. Cell Dev. Biol. 8, 178. 10.3389/fcell.2020.00178 32266260 PMC7096382

[B2] Ahmed R. E. Chanthra N. Anzai T. Koiwai K. Murakami T. Suzuki H. (2021). Sarcomere shortening of pluripotent stem cell-derived cardiomyocytes using fluorescent-tagged sarcomere proteins. J. Vis. Exp. 10.3791/62129 33749676

[B3] Ahmed R. E. Tokuyama T. Anzai T. Chanthra N. Uosaki H. (2022). Sarcomere maturation: function acquisition, molecular mechanism, and interplay with other organelles. Philos. Trans. R. Soc. Lond B Biol. Sci. 377 (1864), 20210325. 10.1098/rstb.2021.0325 36189811 PMC9527934

[B4] Aleksander S. A. Balhoff J. Carbon S. Cherry J. M. Drabkin H. J. Ebert D. (2023). The Gene ontology knowledgebase in 2023. Genetics 224, iyad031. 10.1093/genetics/iyad031 36866529 PMC10158837

[B5] Andersen P. Tampakakis E. Jimenez D. V. Kannan S. Miyamoto M. Shin H. K. (2018). Precardiac organoids form two heart fields *via* bmp/Wnt signaling. Nat. Commun. 9 (1), 3140. 10.1038/s41467-018-05604-8 30087351 PMC6081372

[B6] Anzai T. Yamagata T. Uosaki H. (2020). Comparative transcriptome landscape of mouse and human hearts. Front. Cell Dev. Biol. 8, 268. 10.3389/fcell.2020.00268 32391358 PMC7188931

[B7] Ashburner M. Ball C. A. Blake J. A. Botstein D. Butler H. Cherry J. M. (2000). Gene ontology: tool for the unification of biology. Nat. Genet. 25 (1), 25–29. 10.1038/75556 10802651 PMC3037419

[B8] Cardoso-Moreira M. Halbert J. Valloton D. Velten B. Chen C. Shao Y. (2019). Gene expression across mammalian organ development. Nature 571 (7766), 505–509. 10.1038/s41586-019-1338-5 31243369 PMC6658352

[B9] Chanthra N. Abe T. Miyamoto M. Sekiguchi K. Kwon C. Hanazono Y. (2020). A novel fluorescent reporter system identifies Laminin-511/521 as potent regulators of cardiomyocyte maturation. Sci. Rep. 10 (1), 4249. 10.1038/s41598-020-61163-3 32144297 PMC7060274

[B10] Chirikian O. Goodyer W. R. Dzilic E. Serpooshan V. Buikema J. W. McKeithan W. (2021). CRISPR/Cas9-based targeting of fluorescent reporters to human iPSCs to isolate atrial and ventricular-specific cardiomyocytes. Sci. Rep. 11 (1), 3026. 10.1038/s41598-021-81860-x 33542270 PMC7862643

[B11] Dobin A. Davis C. A. Schlesinger F. Drenkow J. Zaleski C. Jha S. (2013). STAR: ultrafast universal RNA-seq aligner. Bioinformatics 29 (1), 15–21. 10.1093/bioinformatics/bts635 23104886 PMC3530905

[B12] Engler J. B. (2025). Tidyplots empowers life scientists with easy code‐based data visualization. iMeta 4 (2), e70018. 10.1002/imt2.70018 40236782 PMC11995173

[B13] Gentillon C. Li D. Duan M. Yu W. M. Preininger M. K. Jha R. (2019). Targeting HIF-1α in combination with PPARα activation and postnatal factors promotes the metabolic maturation of human induced pluripotent stem cell-derived cardiomyocytes. J. Mol. Cell. Cardiol. 132, 120–135. 10.1016/j.yjmcc.2019.05.003 31082397 PMC6683286

[B14] Guo Y. Pu W. T. (2020). Cardiomyocyte maturation: new phase in development. Circ. Res. 126 (8), 1086–1106. 10.1161/CIRCRESAHA.119.315862 32271675 PMC7199445

[B15] Guo Y. Jardin B. D. Zhou P. Sethi I. Akerberg B. N. Toepfer C. N. (2018). Hierarchical and stage-specific regulation of murine cardiomyocyte maturation by serum response factor. Nat. Commun. 9 (1), 3837. 10.1038/s41467-018-06347-2 30242271 PMC6155060

[B16] Kamakura T. Makiyama T. Sasaki K. Yoshida Y. Wuriyanghai Y. Chen J. (2013). Ultrastructural maturation of human-induced pluripotent stem cell-derived cardiomyocytes in a long-term culture. Circ. J. 77 (5), 1307–1314. 10.1253/circj.cj-12-0987 23400258

[B17] Kung A. L. Zabludoff S. D. France D. S. Freedman S. J. Tanner E. A. Vieira A. (2004). Small molecule blockade of transcriptional coactivation of the hypoxia-inducible factor pathway. Cancer Cell 6 (1), 33–43. 10.1016/j.ccr.2004.06.009 15261140

[B18] Liao Y. Smyth G. K. Shi W. (2014). featureCounts: an efficient general purpose program for assigning sequence reads to genomic features. Bioinformatics 30 (7), 923–930. 10.1093/bioinformatics/btt656 24227677

[B19] Maria Cherian R. Prajapati C. Penttinen K. Häkli M. Koivisto J. T. Pekkanen-Mattila M. (2023). Fluorescent hiPSC-derived MYH6-mScarlet cardiomyocytes for real-time tracking, imaging, and cardiotoxicity assays. Cell Biol. Toxicol. 39 (1), 145–163. 10.1007/s10565-022-09742-0 35870039 PMC10042918

[B20] Miki K. Deguchi K. Nakanishi-Koakutsu M. Lucena-Cacace A. Kondo S. Fujiwara Y. (2021). ERRγ enhances cardiac maturation with T-tubule formation in human iPSC-derived cardiomyocytes. Nat. Commun. 12 (1), 3596. 10.1038/s41467-021-23816-3 34155205 PMC8217550

[B21] Moriwaki T. Tani H. Haga K. Morita-Umei Y. Soma Y. Umei T. C. (2023). Scalable production of homogeneous cardiac organoids derived from human pluripotent stem cells. Cell Rep. Methods 3 (12), 100666. 10.1016/j.crmeth.2023.100666 38113855 PMC10753388

[B22] Murphy S. A. Miyamoto M. Kervadec A. Kannan S. Tampakakis E. Kambhampati S. (2021). PGC1/PPAR drive cardiomyocyte maturation at single cell level *via* YAP1 and SF3B2. Nat. Commun. 12 (1), 1648. 10.1038/s41467-021-21957-z 33712605 PMC7955035

[B23] Nakada Y. Canseco D. C. Thet S. Abdisalaam S. Asaithamby A. Santos C. X. (2017). Hypoxia induces heart regeneration in adult mice. Nature 541 (7636), 222–227. 10.1038/nature20173 27798600

[B24] Nakano H. Minami I. Braas D. Pappoe H. Wu X. Sagadevan A. (2017). Glucose inhibits cardiac muscle maturation through nucleotide biosynthesis. eLife 6, e29330. 10.7554/eLife.29330 29231167 PMC5726851

[B25] Nishiyama C. Saito Y. Sakaguchi A. Kaneko M. Kiyonari H. Xu Y. (2022). Prolonged myocardial regenerative capacity in neonatal opossum. Circulation 146 (2), 125–139. 10.1161/CIRCULATIONAHA.121.055269 35616010

[B26] Sakamoto T. Matsuura T. R. Wan S. Ryba D. M. Kim J. Won K. J. (2020). A critical role for estrogen-related receptor signaling in cardiac maturation. Circ. Res. 126 (12), 1685–1702. 10.1161/CIRCRESAHA.119.316100 32212902 PMC7274895

[B27] Shinnawi R. Huber I. Maizels L. Shaheen N. Gepstein A. Arbel G. (2015). Monitoring human-induced pluripotent stem cell-derived cardiomyocytes with genetically encoded calcium and voltage fluorescent reporters. Stem Cell Rep. 5 (4), 582–596. 10.1016/j.stemcr.2015.08.009 26372632 PMC4624957

[B28] Thomas P. D. Ebert D. Muruganujan A. Mushayahama T. Albou L. Mi H. (2022). PANTHER: making genome‐scale phylogenetics accessible to all. Protein Sci. 31 (1), 8–22. 10.1002/pro.4218 34717010 PMC8740835

[B29] Uosaki H. Taguchi Y. h. (2016). Comparative gene expression analysis of mouse and human cardiac maturation. Genomics Proteomics Bioinforma. 14 (4), 207–215. 10.1016/j.gpb.2016.04.004 27431744 PMC4996857

[B30] Uosaki H. Cahan P. Lee D. I. Wang S. Miyamoto M. Fernandez L. (2015). Transcriptional landscape of cardiomyocyte maturation. Cell Rep. 13 (8), 1705–1716. 10.1016/j.celrep.2015.10.032 26586429 PMC4662925

[B31] Yamanaka S. Zahanich I. Wersto R. P. Boheler K. R. (2008). Enhanced proliferation of monolayer cultures of embryonic stem (ES) cell-derived cardiomyocytes following acute loss of retinoblastoma. PLoS ONE 3 (12), e3896. 10.1371/journal.pone.0003896 19066628 PMC2588539

[B32] Yang X. Pabon L. Murry C. E. (2014a). Engineering adolescence: maturation of human pluripotent stem cell–derived cardiomyocytes. Circ. Res. 114 (3), 511–523. 10.1161/CIRCRESAHA.114.300558 24481842 PMC3955370

[B33] Yang X. Rodriguez M. Pabon L. Fischer K. A. Reinecke H. Regnier M. (2014b). Tri-iodo-l-thyronine promotes the maturation of human cardiomyocytes-derived from induced pluripotent stem cells. J. Mol. Cell. Cardiol. 72, 296–304. 10.1016/j.yjmcc.2014.04.005 24735830 PMC4041732

