# Supplementary material for: Transcriptome-based maturation assessment revealed pro-maturation transcription factors of cardiomyocytes
Source: Front Bioeng Biotechnol. 2026 Feb 18;14:1717996. doi: 10.3389/fbioe.2026.1717996 (PMC12957250; doi:10.3389/fbioe.2026.1717996)
Supplement: Supplementary file 8 [file DataSheet1.docx]

Supplementary Material

# Supplementary Tables

**Supplementary Table 1.** List of TFs used in this study, including accession number and source. Information regarding secondary assessment.

**Supplementary Table 2.** Lists of top and bottom weighted genes to PC1 submitted to gene ontology analysis and increased and decreased gene ontologies.

# Supplementary Code and Data

**Supplementary Presentation 1.** ZIP compressed R markdown file for reproducing figures.

**Supplementary Data Sheet 2.** ZIP compressed R data file (rds) for mouse ESC-CMs.

**Supplementary Data Sheet 3.** ZIP compressed R data file (rds) for human hearts and iPSC-CMs.

**Supplementary Data Sheet 4.** ZIP compressed R data file (rds) for mouse ESC-CMs transduced with TFs.

**Supplementary Data Sheet 5.** ZIP compressed R data file (rds) for mouse heart reference data.

# Supplementary Figure

**Supplementary Figure 1.** ESC-CMs with and without thyroid hormone (T3) for up to 14 days.

**
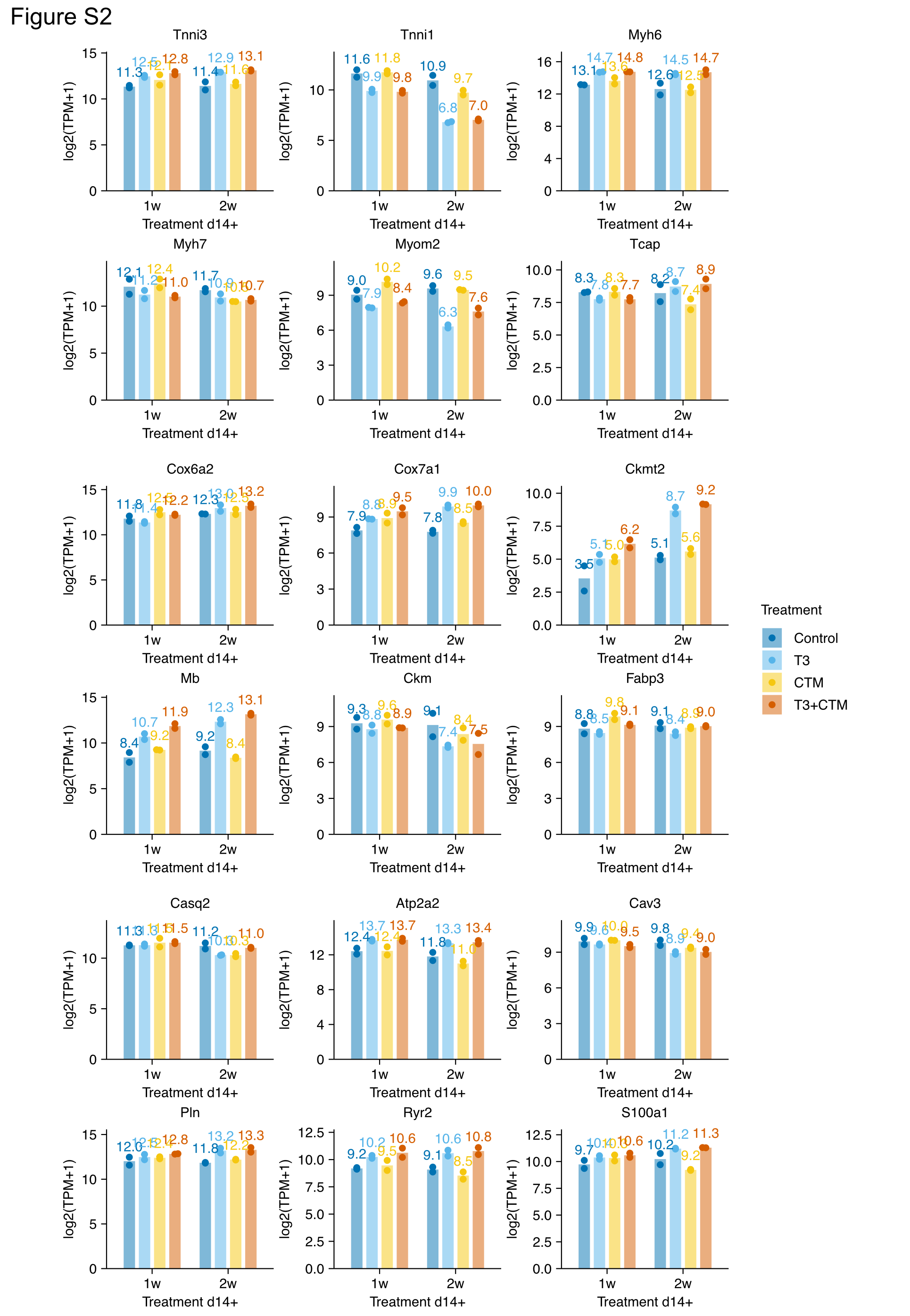
**

**Supplementary Figure 2.** Expressions of maturation-related genes in ESC-CMs with T3/CTM treatment for one and two weeks.

**Supplementary Figure 3.** Endogenous and transgene expression with TF transduction. Wilcoxon test was used. *, p < 0.05; **, p < 0.01.

**Supplementary Figure 4.** Expressions of maturation-related genes. (a) Maturation-related sarcomere gene expressions with selected TFs. (b) Maturation-related metabolic gene expressions with selected TFs. (c) Maturation-related calcium handling gene expressions with selected TFs. Wilcoxon test was used. *, p < 0.05; **, p < 0.01.

**Supplementary Figure 5.** Expression changes of oxidative phosphorylation hallmark genes. Wilcoxon test was used. *, p < 0.05; **, p < 0.01.
